# Mood disorders in adults with epilepsy: a review of unrecognized facts and common misconceptions

**DOI:** 10.1186/s12991-024-00493-2

**Published:** 2024-03-04

**Authors:** Andres M. Kanner, Rohit Shankar, Nils G. Margraf, Bettina Schmitz, Elinor Ben-Menachem, Josemir W. Sander

**Affiliations:** 1 Epilepsy Division and Department of Neurology, Miller School of Medicine, University of Miami, 1120 NW, 14th Street, Room 1324, Miami, FL 33136 USA; 2 University of Plymouth Peninsula School of Medicine, Truro, UK; 3 Cornwall Partnership NHS Foundation Trust, Truro, UK; 4 Department of Neurology, Christian-Albrechts University of Kiel and University Medical Center Schleswig-Holstein, Kiel, Germany; 5 Department of Neurology, Vivantes Humboldt-Klinikum, Berlin, Germany; 6 Department of Clinical Neuroscience, Institute of Neuroscience and Physiology, University of Gothenburg, Gothenburg, Sweden; 7 Department of Clinical and Experimental Epilepsy, UCL Queen Square Institute of Neurology, London, WC1N 3BG UK; 8 Stichting Epilepsie Instellingen Nederland (SEIN), Heemstede, 2103SW The Netherlands; 9 Chalfont Centre for Epilepsy, Chalfont St Peter, UK; 10 Department of Neurology, West China Hospital of Sichuan University, Chengdu, China

**Keywords:** Depression, Suicidality, Pharmacological interactions

## Abstract

Epilepsy is one of the most common neurologic conditions. Its clinical manifestations are not restricted to seizures but often include cognitive disturbances and psychiatric disorders. Prospective population-based studies have shown that people with epilepsy have an increased risk of developing mood disorders, and people with a primary mood disorder have an increased risk of developing epilepsy. The existence of common pathogenic mechanisms in epilepsy and mood disorders may explain the bidirectional relation between these two conditions. Recognition of a personal and family psychiatric history at the time of evaluation of people for a seizure disorder is critical in the selection of antiseizure medications: those with mood-stabilizing properties (e.g., lamotrigine, oxcarbazepine) should be favoured as a first option in those with a positive history while those with negative psychotropic properties (e.g., levetiracetam, topiramate) avoided. While mood disorders may be clinically identical in people with epilepsy, they often present with atypical manifestations that do not meet ICD or DSM diagnostic criteria. Failure to treat mood disorders in epilepsy may have a negative impact, increasing suicide risk and iatrogenic effects of antiseizure medications and worsening quality of life. Treating mood disorders in epilepsy is identical to those with primary mood disorders. Yet, there is a common misconception that antidepressants have proconvulsant properties. Most antidepressants are safe when prescribed at therapeutic doses. The incidence of seizures is lower in people randomized to antidepressants than placebo in multicenter randomized placebo-controlled trials of people treated for a primary mood disorder. Thus, there is no excuse not to prescribe antidepressant medications to people with epilepsy.

## Introduction

Epilepsy is one of the most common neurologic disorders affecting all ages. Its clinical manifestations are not restricted to seizures [1] but often include cognitive disturbances and comorbid psychiatric disorders, which must be identified and treated. Mood and anxiety disorders are the two most frequent psychiatric comorbidities diagnosed in up to a third of people with epilepsy [2]. The cause of mood disorders (MD) in epilepsy is multifactorial and results from genetic, neurobiological, and psychosocial factors [3]. Epilepsy and its comorbid MD represent a global issue in terms of mental health, which was highlighted by the WHO Mental Health Action Plan 2013–2030 [4].

Mood disorders affect people with epilepsy at several levels: (i) they impact management as they are associated with an increased risk of treatment-resistant epilepsy and of a higher incidence of iatrogenic psychiatric and non-psychiatric adverse events associated with antiseizure medications (ASMs) [5, 6]; (ii) they are associated with an increased risk of premature mortality, resulting from a higher occurrence of completed suicide, and of death associated with other external causes [7]; (iii) they increase costs to individuals, families and society through greater use of medical services [8]; (iv) among people with treatment-resistant focal epilepsy, depressive symptoms are one of the leading independent predictors of poor quality of life more than seizure frequency and severity [9]. Mood disorders can also negatively impact people with reasonable seizure control and in an advantaged socioeconomic situation [10]. This narrative review identifies the unrecognized facts and common misconceptions in managing adults with mood disorders and epilepsy. We have conducted a literature search in PubMed using the keywords “Epilepsy”, “Adult” and “Mood Disorders” and focused but were not limited to the last 5 years.

### Epidemiology of mood disorders in epilepsy

Mood disorders in epilepsy encompass discrete conditions such as dysthymia, major depressive and bipolar disorders, and atypical presentations such as Interictal Dysphoric Disorder [3, 11, 12]. In many studies, however, investigators relied solely on the presence of "symptoms of depression" identified with screening instruments, which were reported as a manifestation of "depression in epilepsy", but which may not require the use of pharmacotherapy. Such symptoms could have expressed any categorical diagnosis, a sub-syndromic MD, or a reactive depression. Consequently, the prevalence rates of reported MD in people with epilepsy have ranged between 20 and 50%, depending on the method of ascertainment (self-report vs. screening tools vs. structured and unstructured clinical interviews), type of population investigated and of MD identified. For example, in one Canadian population-based study, investigators used a structured interview to determine the lifetime prevalence of categorical diagnoses of mood and anxiety disorders in epilepsy based on the DSM-IV diagnostic criteria [13]. They found a lifetime prevalence of major depressive disorder of 17.4% (95% CI:10.0–24.9), of any MD, 24.4% (95% CI: 16.0–32.8), and of any MD, including dysthymia of 34.2% (25.0–43.3).

In contrast, a meta-analysis of population-based studies of over a million adults reported a cross-point prevalence of "depression" in epilepsy of about 25% with an overall odds ratio of 2.7 compared with the general population [14], while another meta-analysis that included 27 studies and over 3000 individuals, showed a pooled prevalence of 20.2% [15]. The diagnosis of "depression" reflected heterogeneous data, many of which relied on self-rating instruments that identify "symptoms of depression" and not a categorical diagnosis of one of the MDs cited above. Additional research is needed to establish the prevalence rates of the various types of MD in epilepsy. Nonetheless, there is a consensus that higher prevalence rates occur among those with treatment-resistant epilepsy [3, 6].

### The bidirectional relations between epilepsy and MD

Prospective population-based studies have shown a bidirectional relation between epilepsy and MD, as people with epilepsy have an increased risk of developing MD and people with a primary MD have an increased risk of developing epilepsy. For example, in a large U.K. observational cohort study, a history of an MD was associated with a 2.5-fold increased risk of developing epilepsy [16]. Likewise, in a large population-based study, Adelow et al. showed an odds ratio (OR) for unprovoked seizures of 2.5 (1.7–3.7) after a hospital discharge diagnosis for depression, 2.7 (1.4–5.3) for bipolar disorder, 2.3 (1.5–3.5) for psychosis, 2.7 (1.6–4.8) for anxiety disorders, and 2.6 (1.7–4.1) for suicide attempts [17]. Furthermore, the risk of developing unprovoked epileptic seizures was highest less than 2 years before and up to 2 years after a first psychiatric diagnosis. Conversely, those with epilepsy had a two- to three-fold higher risk of developing an MD [16]. Suicide risk was increased almost threefold in epilepsy, while suicidality preceding a diagnosis of epilepsy was associated with a fourfold higher risk of developing epilepsy [16]. A review suggested that the overall suicidal rate in epilepsy is over five times higher than in the general population. Specifically, in people with temporal lobe epilepsy, it was 25-fold higher [18, 19]. A review of 12 studies assessing causes of death by suicide in people with epilepsy found that suicide accounted for death in 12% compared to 1.1–1.2% in the general population [20].

The existence of common pathogenic mechanisms in epilepsy and MD has been suggested as a possible explanation for the bidirectional relation between these two conditions. The common pathogenic mechanisms include (i) abnormal neurotransmitter function of serotonin (5HT), norepinephrine (NE), dopamine (DA), glutamate and gamma-amino-butyric acid (GABA); (ii) neuro-endocrine disturbances such as a hyperactive-hypothalamic pituitary adrenal axis resulting in increased cortisol secretion; (iii) abnormal neuroinflammatory processes, including increased secretion of cytokines [21–23].

5HT transmission deficits in human depression seem to be partially related to a lower density of serotonergic innervation in terminal areas and a deficiency in serotonin transporter binding sites in the postmortem human brain [21]. A decreased density or affinity of postsynaptic 5HT1A receptors was found in the hippocampus and amygdala of people with untreated depression dying from suicide. A link to defects in the dorsal raphe nuclei with an impaired serotonergic transmission resulting in an excessive density of serotonergic somatodendritic impulse suppressing 5HT1A autoreceptors in depressed suicide victims has also been postulated. [22]. The role of 5HT has been investigated in humans with temporal lobe epilepsy (TLE) with positron emission tomography studies targeting the 5HT1A receptor [23]. In people with and without hippocampal atrophy, a reduced 5HT1A receptor binding capacity was present in mesial temporal areas ipsilateral to the seizure focus. A 20% binding decrease was found in the raphe and a third lower binding in the ipsilateral thalamic region to the seizure focus [23]. It showed that epileptogenic hippocampus, amygdala, anterior cingulate, and lateral temporal neocortex ipsilateral to the seizure focus, contralateral hippocampi and raphe nuclei all presented a decreased binding. A decreased 5HT1A receptor binding was also found in the amygdala, hippocampus, cingulate gyrus, and raphe nucleus of people with primary MD. Anatomical changes identified with high-resolution MRI and volumetric measurements, such as temporal and frontal lobes atrophy, were also seen in people with primary MD, bipolar disorders, and epilepsy [24]. Whether the bidirectional relation is more likely to occur in people with focal epilepsy of frontal and/or temporal origin is yet to be established. The bidirectional link between MD and epilepsy was also suggested by data from a study that compared seizure incidence between subjects with a primary MD randomized to an antidepressant drug and placebo in Phase II and III clinical trials of several selective serotonin reuptake inhibitors (SSRIs), venlafaxine and the a2-antagonist mirtazapine [25]. The seizure incidence was significantly lower among people randomized to antidepressants. In comparison, those randomized to placebo experienced a 19-fold higher incidence of unprovoked seizures than expected in the general population. These data may also suggest a possible protective effect against seizures of SSRIs and serotonin and norepinephrine reuptake inhibitors SNRIs [25], but this has yet to be established in double-blind placebo-controlled trials.

Increased glutamate and decreased GABA concentrations have been reported in magnetic resonance spectrometry-1 studies in people with epilepsy and those with MD [26]. An increased secretion of cytokines, including TNF-alpha, interleukin 1beta, and interleukin-6 has been reported in people with primary MD and epilepsy [27].

### Clinical manifestations of depression in epilepsy

Mood disorders in people with epilepsy may be identical to the primary mood disorders and present with major depressive disorders, bipolar disorders, dysthymia, cyclothymia. These meet the diagnostic criteria of the Diagnostic and Statistical Manual of Mental Disorders 5-TR (DSM-V-TR) [28] and will not be reviewed here. MD in people with epilepsy may also have atypical clinical presentations that do not meet diagnostic criteria included in the DSM and International Classification of Diseases (ICD-11). These include interictal dysphoric disorder (IDD) and peri-ictal depressive episodes [11, 12, 29, 30].

Interictal dysphoric disorder is seen in approximately 30 to 50% of depressed people with epilepsy [12, 30]. They consist of symptoms of depression intermixed with brief euphoric moods, irritability, anxiety, paranoid feelings, and somatoform symptoms (anergia, atypical pain, and insomnia). They tend to follow a chronic course with recurrent symptom-free periods, primarily affecting people with treatment-resistant epilepsy.

Peri-ictal depressive episodes may precede (pre-ictal), be the expression of the ictus (ictal) or follow seizures (postictal). This period is recognized as the peri-ictal period [29, 30]. The most commonly recognized peri-ictal depressive episodes are postictal and tend to occur in people with treatment-resistant epilepsy [29–31]. Preictal depressive episodes typically present as a dysphoric mood. The symptoms may extend for hours or even one to three days before the onset of a seizure and remit completely the day after the ictus.

Only a few studies have investigated postictal symptoms of depression, the largest of which included 100 consecutive people with treatment-resistant focal epilepsy [29]. Postictal depressive symptoms were identified in 43 of them. These symptoms occurred after more than 50% of seizures during the previous three months, and their duration ranged from 0.5 to 108 h, with a median time of 24 h. These symptoms could occur within 72 h of the last seizure, and typically, a symptom-free period of 12 to 24 h between the seizure and the onset of the psychiatric symptoms was recorded. Postictal suicidal ideation was identified in 13% of them, and the median duration of these symptoms was also 24 h.

Ictal depressive episodes are the clinical expression of a focal aware seizure in which the depressive symptoms are the sole (or predominant) semiology [6]. The most frequent symptoms include feelings of anhedonia, guilt, and suicidal ideation. Such mood changes are typically brief, stereotypical, occur out of context, and are associated with other ictal phenomena. More commonly, however, ictal symptoms of depression are followed by an alteration of consciousness as the ictus evolves from a focal aware to a focal unaware seizure.

Peri-ictal psychiatric symptoms are often unrecognized despite their relatively high prevalence in people with treatment-resistant epilepsy [29–31]. DSM and ICD classification schemes do not recognize peri-ictal depressive episodes temporally related to epileptic seizures. The ICD-11 codes to identify psychiatric disorders in epilepsy are essentially not applicable as ICD codes have low sensitivity for some psychiatric conditions, leading to possible underestimation of their actual prevalence.

### Suicidality in epilepsy

Suicidality comprises suicidal ideation (SI), suicidal behaviour (e.g., attempts) and completed suicide. Suicidality has been reported in epilepsy with a higher frequency than in the general population [19, 32, 33]. People with epilepsy have a twofold higher risk of attempting and completing suicide even without presenting with psychiatric comorbidity [7]. When a diagnosis of drug and alcohol abuse, mood disorder, anxiety and personality disorder coexist, the risk of completed suicide increases up to 32-fold [7]. SI and behaviour are often unrecognized until too late.

Suicidality and epilepsy have a complex relationship involving some shared pathogenic mechanisms. SI and suicidal behaviour can express interictal, peri-ictal, and iatrogenic phenomena. Interictal SI and suicidal behaviour are independent of seizures, while postictal SI and suicidal behaviour can express postictal depressive and /or psychotic episodes [19, 29].

The Columbia Classification Algorithm of Suicide Assessment (C-CASA) is a standardized tool to measure the clinical manifestations of suicidality [34], as reported in Table 1.

**Table 1:** C-CASA definitions

Classification/category	Definition
Suicidal events Completed suicide	A self-injurious behaviour that resulted in fatality and was associated with at least some intent to die as a result of the act
Suicide attempt	A potentially self-injurious behaviour associated with at least some intent to die due to the act. Evidence that the individual intended to kill themself, at least to some degree, can be explicit or inferred from the behaviour or circumstance. A suicide attempt may or may not result in actual injury
Preparatory acts toward imminent suicidal behaviour	The individual takes steps to injure him- or herself but is stopped by self or others from starting the self-injurious act before the potential for harm has begun
Suicidal ideation	Passive thoughts about wanting to be dead or active thoughts about killing oneself, not accompanied by preparatory behavior^a^
Nonsuicidal events self-injurious behaviour, no suicidal intent	Self-injurious behaviour associated with no plan to die. The behaviour is intended purely for other reasons, either to relieve distress (often referred to as "self-mutilation", e.g., superficial cuts or scratches, hitting/ banging, or burns) or to effect change in others or the environment
Other, no deliberate self-harm	No evidence of any suicidality or deliberate self-injurious behaviour associated with the event. The event is characterized as an accidental injury, psychiatric or behavioural symptoms only, or medical symptoms or procedure only
Indeterminate or potentially suicidal events self-injurious behaviour, suicidal intent unknown	Self-injurious behaviour where associated intent to die is unknown and cannot be inferred. The injury or potential for damage is clear, but why the individual engaged in that behaviour is unclear
Not enough information	Insufficient information to determine whether the event involved deliberate suicidal behaviour or ideation. There is reason to suspect the possibility of suicidality but not enough to be confident that the event was not something other, such as an accident or psychiatric symptom. An injury sustained on a place on the body consistent with deliberate self-harm or suicidal behaviour (e.g., wrists), with- out any information as to how the injury was received, would warrant placement in this category

^a^If ideation is deemed inherently related to a behavioural act, a separate rating is not given. However, if there is no clear relationship to a behavioural event, a separate classification od ideation is warranted

The incidence risk ratio of suicide is elevated for three years prior (3.1–4.5) and the first year following the diagnosis of epilepsy [16]. These data express a bidirectional relation between suicidality and epilepsy in which people with epilepsy are at increased risk of developing SI and behaviour. In contrast, people with suicidality have a higher risk of developing epilepsy. Common pathogenic mechanisms in epilepsy and suicidality are hypothesized to explain this bidirectional relation [35].

Screening for suicidality should be essential to evaluating epilepsy, given the relatively high prevalence of SI and the increased risk of complete suicide observed in this setting. In addition to identifying suicidality symptoms, such screening must include the common psychiatric comorbidities associated with epilepsy, such as MD and anxiety disorders [32]. When applied to everyday clinical practice, self-reporting screening instruments can facilitate the screening of suicidality and these psychiatric comorbidities. The Neurological Disorders Depression Inventory for Epilepsy (NDDIE) [36] is the preferred instrument to screen for major depressive episodes, specifically for people with epilepsy. Suicidality can be screened using item 4 of the NDDIE (‘I would be better off dead’) as it has excellent discrimination and specificity, reliable sensitivity, and positive and negative likelihood ratios. Alternatively, clinicians can rely on item 9 of the Beck Depression Inventory-II [37], which can provide more details regarding SI's passive or active nature. When research is the goal, the MINI Neuropsychiatric Interview (MINI) can be used to identify comorbid discrete psychiatric diagnoses, SI, and suicidal behaviour. A trained worker must administer this interview [38]. The Generalized Anxiety Disorder-7 is a self-report questionnaire commonly used to screen for GAD and panic disorders [39].

People expressing suicidality should be referred to a mental health professional to identify causes. All interventions should be considered, non-pharmacological and pharmacological, as most psychotropic drugs are safe at recommended doses in epilepsy. For example, the Three-Step Theory of Suicide proposed by Klonsky and May can be implemented to prevent the progression from suicidal ideation to suicidal attempt in patients with risks factors associated with suicidality [40]. Further multidisciplinary research is needed to understand further the epidemiology and risk factors of suicidal behaviour in epilepsy. This interdisciplinary approach involving neurology and psychiatry can lead to robust and validated prediction models and shared evidence-based prevention strategies that will help reduce the risk of undertreatment of MD.

### Iatrogenic causes of MD in epilepsy

Antiseizure medications (ASM) may trigger or worsen psychiatric symptoms, including depression, anxiety, psychosis, and attention deficit hyperactive disorder (ADHD) features. These iatrogenic adverse events are more likely to occur in people with epilepsy:

1. At risk of developing psychiatric disorders (personal or family history of psychiatric disorders) started on ASMs with known negative psychotropic properties (GABAergic and glutamatergic drugs [e.g., barbiturates, topiramate, zonisamide, levetiracetam and perampanel) [41, 42].
2. With a history of primary mood and anxiety disorders which had remitted with ASMs that have anxiolytic (gabapentin, pregabalin, benzodiazepines) or mood stabilizing properties (carbamazepine, oxcarbazepine, lamotrigine, valproic acid) and who may experience a recurrence of the psychiatric disorder upon their discontinuation [6].
3. Undergoing treatment with psychotropic drugs and enzyme-inducing ASMs (carbamazepine, phenytoin, barbiturates, rufinamide, high doses of topiramate and oxcarbazepine), can unmask psychiatric symptoms by increasing the clearance of psychotropic drugs thus limiting their efficacy.

Identifying people with a current, past and/or family psychiatric history is the first step towards reducing iatrogenic psychiatric symptomatology [6]. In 2008, the Food and Drug Administration (FDA) released a warning on “all” ASMs having the potential to cause suicidal ideation and suicidal behaviour. The FDA’s data had methodologic problems, with questions about its validity raised by professional societies, such as the American Epilepsy Society and the American Academy of Neurology [43]. The fact is that some of the ASMs with negative psychotropic properties can cause SI, particularly in people with a prior and /or family psychiatric history. In such cases, the prescription of these ASMs should be carried out with great caution, and individuals and family members should be advised on potential iatrogenic adverse events. Of note, in 2004, the FDA had required that a black box warning against increased suicidal ideation and behaviour associated with all antidepressants be included in their package inserts. The validity of this warning was questioned and caused great concern because it was followed by a decline in the number of prescriptions of these drugs and an increase in suicides in patients with severe MD [44].

### Pharmacological treatment of MD in epilepsy

The most critical obstacle to treating MDs in epilepsy remains failing to recognize them. [3, 6] Current evidence on managing psychiatric comorbidities in epilepsy is limited. The current consensus among experts is using the same protocols for treating primary MD to achieve complete symptom remission.

In 2021, the Task Force of the International League Against Epilepsy (ILAE) Commission on Psychiatry, the ILAE Executive and the International Bureau for Epilepsy (IBE) issued the following recommendations for the treatment of MD [45]: (1) for mild depression, psychological interventions is the recommended first-line treatment, and when medication is required, SSRIs are first-choice medications. (2) SSRIs remain the first-choice medications for moderate to severe depressive episodes, and SNRIs (venlafaxine) should be considered when people fail to achieve remission of the symptoms with an SSRI. If symptoms persist at optimal doses after two trials, one with an SSRI and another with an SNRI. In that case, the individual needs to be referred to a psychiatric service as the possibility of refractory mood disorder is likely. In such cases, augmentation with mood-stabilizing agents like atypical antipsychotic drugs (aripiprazole, quetiapine, or risperidone) can be considered. Antidepressant treatment should be maintained for at least six months following remission from a first depressive episode. Still, it should be prolonged to nine months in people with a history of previous episodes. It should continue even longer in severe depression or cases of residual symptomatology until such symptoms have subsided.

There is evidence that MD in people with epilepsy respond better to pharmacotherapy and cognitive behaviour therapy (CBT) than primary MD. For example, one randomized controlled study compared the safety and efficacy of sertraline vs. CBT in treating MD in people with epilepsy. Remission was reached in nearly 60% of people randomized to both therapies [46]. In contrast, symptom remission in primary MDE is typically achieved by about a third of people after the first trial. Others have made similar observations from data obtained in open trials [47, 48].

The concern that antidepressant drugs are proconvulsant has been a frequent obstacle in the pharmacologic treatment of MD in people with epilepsy. This is a long-held misconception, as most antidepressant medications are safe in people with epilepsy when prescribed at therapeutic doses. A careful review of the evidence has revealed that most cases of seizures associated with antidepressants were seen in the setting of an overdose [6]. In people with primary mood and anxiety disorders who participated in multicenter randomized double-blind placebo-controlled trials of SSRIs, SNRIs, tricyclic antidepressants (TCAs), seizures were significantly lower among individuals randomized to these antidepressants than to placebo [25]. These findings support the bidirectional relation between MD and epilepsy, where people with MD are likelier to develop epilepsy. Failure to recognize these data has also contributed to the misconception that antidepressant drugs have proconvulsant properties. Potential anticonvulsant effects of SSRIs, SNRIs and TCAs have been suggested in animal models of epilepsy. Still, this effect has yet to be established in double-blind placebo-controlled trials in epilepsy.

### Interactions between ASMs and antidepressants

Psychotropic drug selection must be based on their pharmacokinetic and pharmacodynamic interactions with ASMs.

*Pharmacokinetic interactions:* ASMs can be grouped into those with enzyme-inducing properties, enzyme-inhibiting properties and those that are metabolically inert. The ASMs with enzyme-inducing properties (e.g., carbamazepine, phenytoin, phenobarbital, and primidone) can increase the clearance of other drugs, thus limiting their efficacy. Two ASMs, oxcarbazepine and topiramate, are weak inducers at daily doses over 1200 mg for oxcarbazepine and 200 mg for topiramate. As CYP 450 enzymes also metabolize several psychotropic drugs, their concomitant use with some ASMs can increase clearance and limit their efficacy, lest their dose be adjusted. For example, enzyme-inducing ASMs can lower SSRIs serum concentrations by 25 to 30%. A clinically relevant interaction occurs where carbamazepine, which acts as an inducer, decreases bupropion blood levels by 90% [49, 50].

Conversely, several antidepressant drugs of the SSRI family (fluoxetine and fluvoxamine) can inhibit CYP2C9, thus increasing phenytoin and valproate blood levels. Among the SSRIs, citalopram, escitalopram, and sertraline are usually considered first-line treatments for MD and anxiety disorders, given their lack of pharmacokinetic interaction with ASMs (see below). SNRIs have not been found to have a pharmacokinetic effect on ASMs.

*Pharmacodynamic interactions:* Pharmacodynamic interactions between psychotropic drugs and ASMs can result in iatrogenic side effects. Thus, hyponatremia, often associated with carbamazepine, oxcarbazepine and eslicarbazepine, can be worsened with the concomitant use of several SSRIs and SNRIs. Serum sodium levels must be monitored when these drugs are used together [6]. Likewise, SSRIs can enhance the negative impact on bone health caused by ASMs with enzyme-inducing properties, which can cause osteopenia and osteoporosis. As SSRIs and SNRIs can increase the risk of bleeding, caution should be taken in people with epilepsy taking ASMs with thrombogenic properties like valproic acid. Weight gain associated with several psychotropic drugs (antipsychotic medications, several SSRIs) can be exacerbated by ASMs with similar iatrogenic effects (e.g., valproic acid). Lastly, sexual adverse events are reported in 20 to 30% of people taking SSRIs and SNRIs and can worsen the libido and sexual dysfunction associated with epilepsy and some ASMs [6]. Bupropion is one of the antidepressant drugs with the most negligible impact on sexual functions. Still, its association with an increased risk of seizures leads clinicians to avoid its use in people with epilepsy. Proconvulsant properties in primary MD have been reported with the immediate-release formulation at doses above 300 mg /day, but not with the extended-release formulation.

Antipsychotic drugs with mood-stabilizing properties often used in treating MD have, in general, the potential to increase QT interval. Among these, ziprasidone has the highest risk of affecting QT interval. In addition, people with a prolonged QT may be at increased risk of sudden death. They should have an electrocardiogram performed before commencing antipsychotic therapy and once the target dose is reached [50].

## Conclusions

In an ideal world, people with epilepsy with psychiatric comorbidity should be referred to a mental health professional who would undertake the optimum care tailored to their needs, psychiatric history, disease characteristics and genetics and work with the neurologist to manage the individual’s needs holistically. Unfortunately, access to psychiatric care is often limited, and it frequently falls upon the neurologist to provide pharmacotherapy for their MD. The high prevalence of MD in people with epilepsy, often present by the time of the first recognized epileptic seizure, calls for screening psychiatric comorbidities in the initial stages of their evaluation to encompass all aspects of the individuals’ disease and comorbidities. Furthermore, depressive episodes and symptoms may occur associated with other psychiatric comorbidities that are relatively frequent in patients with epilepsy, including anxiety, attention deficit and psychotic disorders and less frequently, personality disorders. These data are essential in the management of epilepsy as they will dictate the selection of the ASM to avoid iatrogenic psychiatric effects or provide therapeutic effects to comorbid MD, improve the tolerance of ASM, minimize the increased suicidality risk and improve the quality of life of people with epilepsy. Neurologists can be expected to treat MDE, dysthymia and their comorbid anxiety disorders. They are not expected to manage all types of MD, such as bipolar disorders, people with suicidal ideation and behaviour and MD with psychotic features.

## Data Availability

Data sharing does not apply as no datasets were generated or analysed during the current study.

## Abbreviations

ADHD: Attention deficit hyperactive disorder
ASMs: Antiseizure medications
CBT: Cognitive behaviour therapy
C-CASA: Columbia Classification Algorithm of Suicide Assessment
DA: Dopamine
DSM-V-TR: Diagnostic and Statistical Manual of Mental Disorders 5-TR
FDA: Food and Drug Administration
GABA: Gamma-amino-butyric acid
GAD: Generalized anxiety disorder
5HT: 5-Hydroxy-triptamine
IBE: International Bureau for Epilepsy
ICD-11: International Classification of Diseases 11
ILAE: International League Against Epilepsy
MD: Mood disorders
NDDIE: Neurological Disorders Depression Inventory for Epilepsy
NE: Norepinephrine
SCID: Structured Clinical Interview for DSM-IV
SI: Suicidal ideation
SNRIs: Serotonin and norepinephrine reuptake inhibitors
SSRIs: Selective serotonin reuptake inhibitors
TCAs: Tricyclic antidepressants
TLE: Temporal lobe epilepsy
